# Comparison the effects of finerenone and SGLT2i on cardiovascular and renal outcomes in patients with type 2 diabetes mellitus: A network meta-analysis

**DOI:** 10.3389/fendo.2022.1078686

**Published:** 2022-12-15

**Authors:** Xuefeng Li, Hongli Wu, Huifang Peng, Hongwei Jiang

**Affiliations:** ^1^ Department of Endocrinology and Metabolism, The Second Affiliated Hospital of Henan University of Science and Technology, Luoyang, China; ^2^ Endocrinology and Metabolism Center, The First Affiliated Hospital, and College of Clinical Medicine of Henan University of Science and Technology; Henan Key Laboratory of Rare Diseases, Luoyang, China

**Keywords:** finerenone (BAY 94-8862), SGLT2i, T2DM, cardiovascular and renal outcomes, network meta-analysis

## Abstract

**Background:**

Finerenone and sodium-glucose cotransporter 2 inhibitors (SGLT2i) have been shown to improve cardiovascular and renal outcomes in patients with type 2 diabetes mellitus (T2DM), while the relative efficacy has not been determined.

**Methods:**

The databases of PubMed, Embase and Cochrane were searched for relevant cardiovascular or renal outcome trials of SGLT2i or finerenone. The end points were major adverse cardiovascular events (MACE), nonfatal stroke (NS), myocardial infarction (MI), hospitalization for heart failure (HHF), cardiovascular death (CVD), and renal composite outcome (RCO). Network meta-analysis was performed using Bayesian networks to obtain pooled hazard ratios (HR) and 95% confidence intervals (CI). The probability values for ranking active and placebo interventions were calculated using cumulative ranking curves.

**Results:**

1024 articles were searched, and only 9 studies were screened and included in this meta-analysis with 71793 randomized participants. Sotagliflozin (HR 0.72 95%CI 0.59-0.88, SUCAR=0.93) and canagliflozin (HR 0.80 95%CI 0.67-0.97, SUCAR=0.73) can significantly reduce the risk of MACE compared with placebo. Canagliflozin (HR 0.64 95%CI 0.48-0.86, SUCAR=0.73), sotagliflozin (HR 0.66 95%CI 0.50-0.87, SUCAR=0.69) and empagliflozin (HR 0.65 95%CI 0.43-0.98, SUCAR=0.68) can significantly reduce the risk of HHF compared with placebo. Empagliflozin (HR 0.62 95%CI 0.43-0.89, SUCAR=0.96) can significantly reduce the risk of CVD compared with placebo. Empagliflozin (HR 0.61 95%CI 0.39-0.96, SUCAR=0.74), canagliflozin (HR 0.66 95%CI 0.46-0.92, SUCAR=0.63), and dapagliflozin (HR 0.53 95%CI 0.32-0.85, SUCAR=0.88) can significantly reduce the risk of RCO compared with placebo. Finerenone has reduced the risk of MACE, MI, HHF, CVD and RCO to varying degrees, but they do not show significant difference from placebo and each SGLT2i.

**Conclusion:**

Both SGLT2i and finerenone could reduce the risk of MACE, HHF, MI, CVD, RCO. Finerenone has no obvious advantage than SGLT2i on the effects of cardiovascular and renal protective.

**Systematic review registration:**

https://www.crd.york.ac.uk/PROSPERO/, identifier CRD42022375092.

## Introduction

Type 2 diabetes mellitus (T2DM) is a complex chronic metabolic disease, and according to the World Health Organization (WHO), approximately 425 million adults around the world (8.4% of the world’s adult population) currently are with T2DM (1), which is projected to exceed 700 million adults (9.9% of the World’s adult population) by 2045 year (2). People with T2DM have a twice to three times increased risk of developing cardiovascular disease, which is further increased when chronic kidney injury presents (3). In addition to atherosclerotic cardiovascular disease, patients with T2DM have an increased risk of diabetic kidney disease (DKD) and heart failure (HF) (4).

Sodium-glucose cotransporter 2 inhibitors (SGLT2i) (ie, empagliflozin, canagliflozin, dapagliflozin, ertugliflozin, sotagliflozin) increase urinary glucose excretion by inhibiting glucose reabsorption in renal proximal tubules, resulting in weight loss, improvement of hyperuricemia, lipids, and blood pressure (5). According to the current data, SGLT2i has been shown to improve renal and cardiovascular outcomes in patients with T2DM, especially those with high risk factors for CVD (6) and chronic kidney disease (CKD) (7). The latest guidelines from the American Diabetes Association (ADA) (8, 9) suggest that SGLT2i can be used as a monotherapy or in combination with other hypoglycemic agents.

Some studies have demonstrated that hyperactivation of the halocorticoid receptor is associated with renal and cardiovascular diseases (10). Finerenone (BAY 94-8862), a novel nonsteroidal selective halocorticoid receptor antagonist, has shown strong anti-inflammatory and antifibrotic effects (11). Finerenone has been shown to reduce urinary albumin-to-creatinine ratio in patients with CKD in clinical trials, and decreased cardiovascular morbidity and mortality in patients with advanced CKD and T2DM in the FIGARO-DKD (12) and FIDELIO-DKD (13) studies.

Current clinical trials have shown that SGLT2i and finerenone have protective effects on cardiovascular and renal outcomes in T2DM, but there is a lack of head-to-head studies between them. Therefore, we conducted a network meta-analysis of clinical studies related to SGLT2i and finerenone to evaluate and compare the effects of SGLT2i and finerenone on cardiovascular and renal outcomes.

## Material and methods

### Literature search strategy

The study was conducted and reported in accordance with prespecified protocols and PRISMA guidelines for systematic reviews and meta-analyses. We conducted a systematic search of three major databases, PubMed, Embase and Cochrane, for randomized clinical trials (RCTS) published from inception to August 10, 2022, independently conducted by two investigators. These databases were extensively searched for suitable clinical studies. The search terms included: “finerenone”, “BAY 94-8862”, “empagliflozin”, “canagliflozin”, “dapagliflozin”, “ertugliflozin”, “sotagliflozin” and “type 2 diabetes mellitus” (**Supplementary Table 1**). Only fully published RCTS were included (abstracts were excluded).

### Inclusion and exclusion criteria

Studies meeting these criteria were considered eligible: 1) patients with T2DM and chronic kidney disease (UACR ≥30 mg/g and eGFR ≤90 mL/min/1.73m²) (age ≥18 years). 2) Oral intervention with any dose of “finerenone, empagliflozin, canagliflozin, dapagliflozin, ertugliflozin, sotagliflozin”. 3) The control group received placebo. 4) Randomized controlled trials published in English. Exclusion criteria were as follows: 1) patients with serum potassium concentration ≥4.8 mmol/L. 2) Patients receiving renal replacement therapies. 3) Glycosylated hemoglobin >12%. 4) Animal experiments; 5) Meta-analysis, review, case report, meeting and letter.

### Data extraction

Two reviewers independently extracted data from the included studies, and disagreements were resolved through consultation with the third reviewer to reach a consensus. Data extracted included first author, time of publication, sex, age, diabetes duration, drug intervention, duration of intervention, body-mass index, and outcome. According to the Cochrane handbook (14), systematic evaluation of intervention measures to explore the risk of bias (detailed list is as follows: random sequence generation (selection bias), allocation concealment (selection bias), blinding of participants and personnel (performance bias), blinding of outcome assessment (detection bias), incomplete outcome data (attrition bias), selective reporting (reporting bias), etc.

### Study endpoints

The primary endpoints were MACE, HHF, MI, CVD and NS. The secondary endpoint was RCO, including new onset of macroalbuminuria, ESRD, decline in renal function. We adopted RCO as they were reported in each trial for the patients with/without albuminuria. When the included studies assessed multiple renal outcomes in a trial, we prioritized those described above except for development of macroalbuminuria, to minimize inconsistency between studies.

### Data synthesis and analysis

Using trial-level data with hazard ratios (HRs) and 95% confidence intervals (CI) extracted from the included studies. A network meta-analysis was performed using the R programming language (R Foundation for Statistical Computing, Vienna, Austria) “gemtc” package (version 1.1-0) with a random effect model. Heterogeneity was assessed by the probability values of I². If I² is 25%, 50%, or 75%, the heterogeneity was low, medium, and high, respectively. In this network meta-analysis, we included only randomized controlled trial, so there was only circumstantial evidence between the various active interventions. Therefore, there was no need to test for inconsistency between direct and indirect evidence. The probability values on the lower surface of the cumulative ranking curve (SUCRA) were calculated, and the active and placebo interventions were ranked according to different cardiorenal endpoints, and the SUCRA values were ranked. Drawing radar plot in R programming language “fmsb” package.

## Results

### Characteristics of the included studies

**Figure 1:**
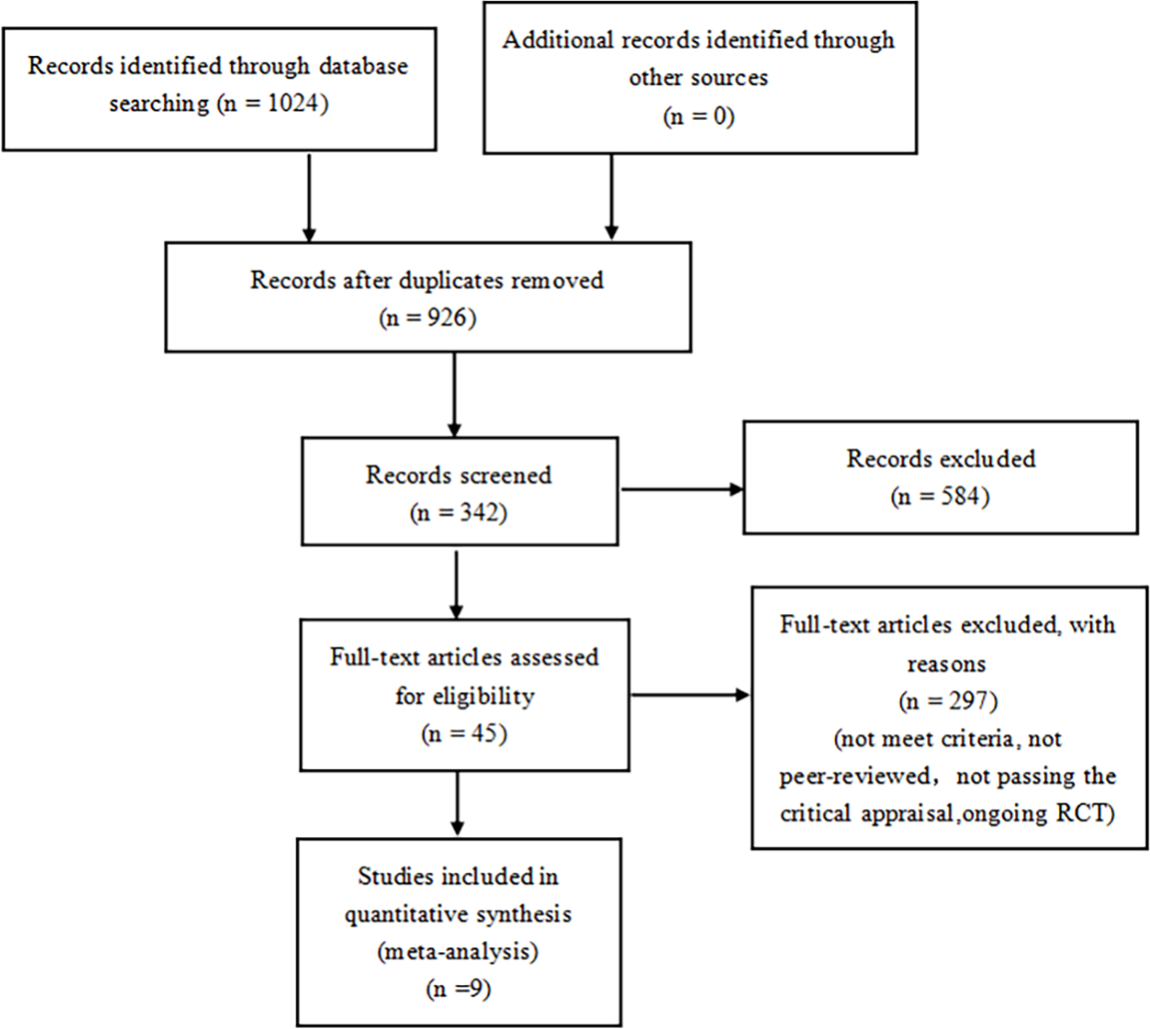
Flow diagram of study selection.

A total of 1024 articles were generated from keywords, eligibility criteria, and databases. Duplicates were removed from the titles and abstracts, 584 were discarded due to topic irrelevance and 324 articles were screened. Of the remaining 45 tests, 23 for not meeting the criteria, 7 were removed because they were not peer-reviewed, 4 for not passing the critical appraisal, 2 were excluded due to ongoing research. 9 studies were reviewed with 71793 randomized participants in total (**Figure 1**). Basic characteristics of studies and participants included in this systematic review and network meta-analysis are presented in **Table 1**. A review of all clinical trials varied in design, population, and primary end points, and search strategies are shown in **Supplementary Table 1**. However, MACE was a common part of every RCTS in the included studies. The summary table in **Supplementary Table 2**.

**Table 1:** The basic characteristics of involved studies.

Study	Years	ID	Study duration (year)	Drugs	Male (%)	Age (year)	HbA1c (%)	Diabetes duration (year)	Body-mass index (kg/m²)
EMPA-REG (15)	2015	NCT01131676	3.1 years	Empagliflozin	71.2	63.1 ± 8.6	8.1 ± 0.8	NA	30.6 ± 5.3
				Placebo	72.0	63.2 ± 8.8	8.1 ± 0.8	NA	30.7 ± 5.2
CANVAS (16)	2017	NCT01032629	3.92 years	Canagliflozin	64.9	63.2 ± 8.3	8.2 ± 0.9	13.5 ± 7.7	31.9 ± 5.9
		NCT01989754		Placebo	63.3	63.4 ± 8.2	8.2 ± 0.9	13.7 ± 7.8	32.0 ± 6.0
CREDENCE (17)	2019	NCT02065791	2.62 years	Canagliflozin	65.4	62.9 ± 9.2	8.3 ± 1.3	15.5 ± 8.7	31.4 ± 6.2
				Placebo	66.7	63.2 ± 9.2	8.3 ± 1.3	16.0 ± 8.6	31.3 ± 6.2
DECLARE–TIMI 58 (18)	2019	NCT01730534	4.2 years	Dapagliflozin	63.1	63.9 ± 6.8	8.3 ± 1.2	11.0	32.1 ± 6.0
				Placebo	62.1	64.0 ± 6.8	8.3 ± 1.2	10.0	32.0 ± 6.1
SOLOIST-WHF (19)	2022	NCT03521934	0.75 years	Sotagliflozin	67.4	63.0-76.0	6.4-8.3	17.0 ± 2.8	30.4
				placebo	65.1	64.0-76.0	6.4-8.2	14.0 ± 2.3	31.1
SCORED (20)	2021	NCT03315143	1.33 years	Sotagliflozin	55.7	69.0 (63.0–74.0)	8.3 (7.6–9.3)	NA	31.9
				placebo	54.5	69.0 (63.0-74.0)	8.0 (7.6-9.4)	NA	31.7
VERTIS CV (21)	2020	NCT01986881	3.5 years	Ertugliflozin	70.3	64.4 ± 8.1	8.2 ± 1.0	12.9 ± 8.3	31.9 ± 5.4
				Placebo	69.3	64.4 ± 8.0	8.2 ± 0.9	13.1 ± 8.4	32.0 ± 5.5
FIGARO-DKD (12)	2022	NCT02545049	3.4 years	Finerenone	68.6	64.1 ± 9.7	7.7 ± 1.4	NA	NA
				Placebo	70.3	64.1 ± 10.0	7.7 ± 1.4	NA	NA
FIDELIO-DKD (13)	2020	NCT02540993	2.6 years	Finerenone	68.9	65.4 ± 8.9	7.7 ± 1.3	16.6 ± 8.8	NA
				Placebo	71.5	65.7 ± 9.2	7.7 ± 1.4	16.6 ± 8.8	NA

NA, Not available.

### Risk of bias assessment

**Figure 2:**
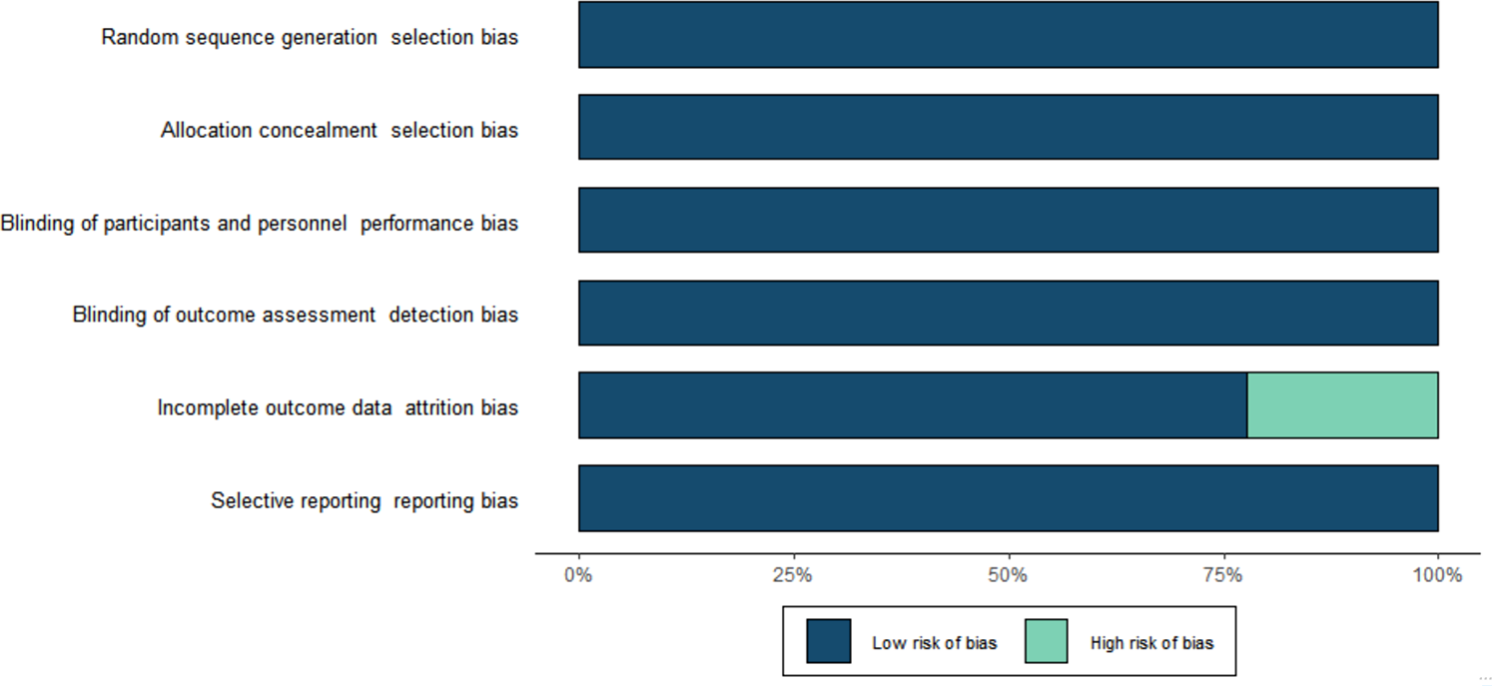
Risk of bias summary.

The risk of bias for all included trials is shown in **Figure 2**. Selection bias was low in all trials. All studies were evaluated as having low performance bias and low detection bias. Attrition and reporting bias were low in all trials. Other sources of bias were high for all included studies because all studies were sponsored by the manufacturer.

### Network meta-analysis of MACE

**Figure 3:**
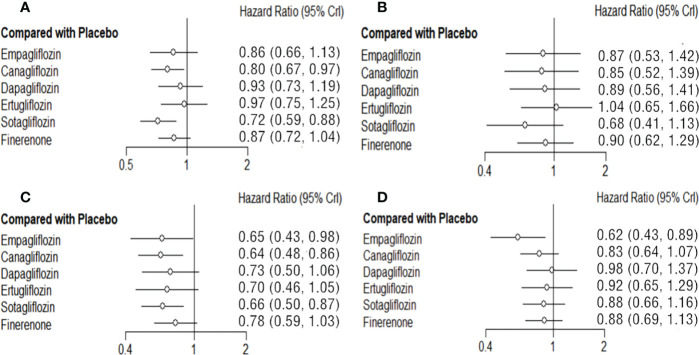
Comparison between the efficacy of SGLT2i and finerenone and placebo on MACE **(A)**, MI **(B)**, HHF**(C)** and CVD **(D)**.

Empagliflozin, canagliflozin, dapagliflozin, ertugliflozin, sotagliflozin and finerenone can reduce the risk of MACE to different degree. Sotagliflozin (vs placebo: HR 0.72 95%CI 0.59-0.88, SUCAR=0.93) shows the best effect, and significantly better than placebo in reducing the risk of MACE, and the tendencies of superior compared with empagliflozin, dapagliflozin, ertugliflozin, canagliflozin, and finerenone do not reach significant difference. Canagliflozin (vs placebo: HR 0.80 95%CI 0.67-0.97, SUCAR=0.73) also shows significantly better result than placebo. The effect of finerenone is slightly inferior to that of sotagliflozin (sotagliflozin vs finerenone: HR 0.83 95%CI 0.63-1.09) and canagliflozin (canagliflozin vs finerenone: HR 0.93 95%CI 0.71-1.20), empagliflozin (empagliflozin vs finerenone: HR 0.99 95%CI 0.72-1.38) without significant difference. And there is also no significant difference about the slightly superiority of finerenon compared with placebo, dapagliflozin and ertugliflozin (**Figure 3A**; **Supplementary Table 3**).

### Network meta-analysis of MI

Empagliflozin, canagliflozin, dapagliflozin, sotagliflozin, and finerenone all reduced the risk of MI to some degrees, while the differences between them or compared with placebo are not significant. Sotagliflozin (vs placebo: HR 0.68 95%CI 0.41-1.13, SUCAR=0.85) is slightly better than others. (**Figure 3B**; **Supplementary Table 4**).

### Network meta-analysis of HHF

Both SGLT2i and finerenone show different good effects on reducing the risk of HHF in T2DM. Compared with placebo, canagliflozin (vs placebo: HR 0.64 95%CI 0.48-0.86, SUCAR=0.73), empagliflozin (vs placebo: HR 0.65 95%CI 0.43-0.98, SUCAR=0.68) and sotagliflozin (vs placebo: HR 0.66 95%CI 0.50-0.87, SUCAR=0.69) show significant advantages. The advantages of ertugliflozin (vs placebo: HR 0.70 95%CI 0.46-1.05, SUCAR=0.56), dapagliflozin (vs placebo: HR 0.73 95%CI 0.50-1.06, SUCAR=0.47), finerenone (vs placebo: HR 0.78 95%CI 0.59-1.03, SUCAR=0.34) show no significant difference compared with placebo. The effect of finerenone on HHF is not significantly different from that of all SGLT2i. (**Figure 3C**, **Supplementary Table 5**).

### Network meta-analysis of CVD

There is significant advantage of empagliflozin (vs placebo: HR 0.62 95%CI 0.43-0.89, SUCAR=0.96) compared with placebo in reducing the risk of CVD. The effects of canagliflozin (vs placebo: HR 0.83 95%CI 0.64-1.07, SUCAR=0.65), dapagliflozin (vs placebo: HR 0.98 95%CI 0.70-1.37, SUCAR=0.28), ertugliflozin (vs placebo: HR 0.92 95%CI 0.65-1.29, SUCAR=0.42) and sotagliflozin (vs placebo: HR 0.88 95%CI 0.66-1.16, SUCAR=0.51) on CVD are not statistical differences. Finerenone (vs placebo: HR 0.88 95%CI 0.69-1.13, SUCAR=0.52) has no significant difference with placebo in reducing the risk of CVD, and compared with SGLT2i, there is still no obvious differences. (**Figure 3D**; **Supplementary Table 6**).

### Network meta-analysis of NS

**Figure 4:**
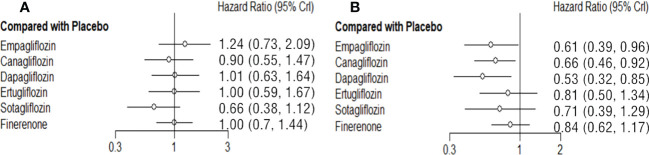
Comparison between the efficacy of SGLT2i and finerenone and placebo on NS **(A)** and RCO **(B)**.

The NS risk reduction functions of each SGLT2i and finerenone are limited compared with placebo. Sotagliflozin (vs placebo: HR 0.66 95%CI 0.38-1.12, SUCAR=0.91) is relatively good, but there is no significant difference. In reducing the risk of NS, there is no significant difference among each SGLT2i and finerenone. (**Figure 4A**; **Supplementary Table 7**).

### Network meta-analysis of treatment groups RCO

Dapagliflozin (vs placebo: HR 0.53 95%CI 0.32-0.85, SUCAR=0.88), empagliflozin (vs placebo: HR 0.61 95%CI 0.39-0.96, SUCAR=0.74), canagliflozin (vs placebo: HR 0.66 95%CI 0.46-0.92, SUCAR=0.63) and are better than placebo in reducing the risk of RCO, and the difference was statistically significant. Ertugliflozin (vs placebo: HR 0.81 95%CI 0.50-1.34, SUCAR=0.36), sotagliflozin (vs placebo: HR 0.71 95%CI 0.39-1.29, SUCAR=0.53) and finerenone (vs placebo: HR 0.84 95%CI 0.62-1.17, SUCAR=0.30) also reduced the risk of RCO, but there is no significant difference compared with placebo. The effects of finerenone and SGLT2i are not significant difference. (**Figure 4B**; **Supplementary Table 8**).

## Discussion

**Figure 5:**
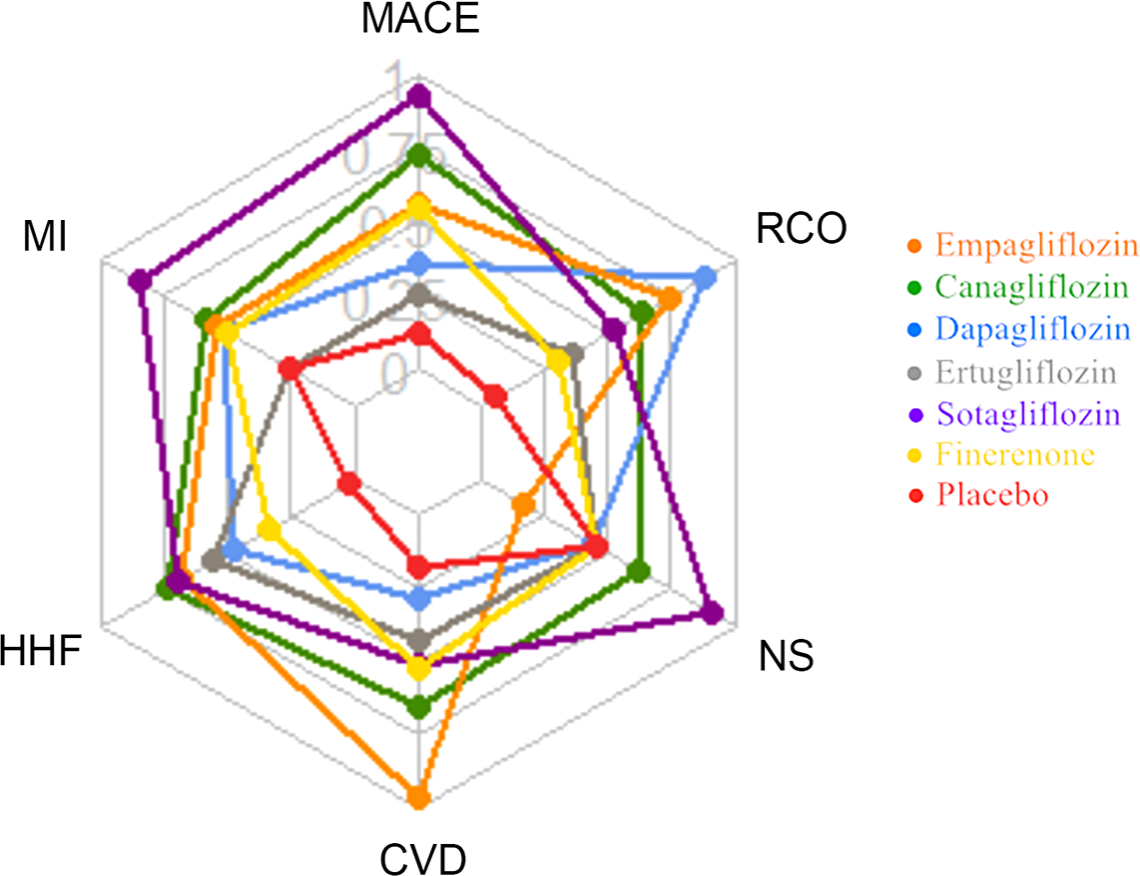
Radar plot of SUCRA values of 6 drugs interventions for cardiorenal and renal outcomes in T2DM patients.

This network meta-analysis included 9 large RCT studies involving 5 different SGLT2i to compare the impact on cardiorenal and renal outcomes with finerenone. In general, SGLT2i significantly improve CV benefits, reduce the risks of MACE, HHF, MI, CVD and other factors compared with placebo, and are also better than finerenone, especially sotagliflozin. Compared with placebo, dapagliflozin, ertugliflozin, empagliflozin, finerenone, reduce the risk of NS has no difference or a slightly elevated, which may be because both SGLT2i and finerenone can reduce the circulating blood volume of subjects through different ways. Compared with placebo, SGLT2i and finerenone have some advantages in improving the renal composite outcome, but SGLT2i is superior to finerenone, especially empagliflozin (**Figure 5**; **Supplementary Table 9**).

Finerenone, a selective nonsteroidal glucocorticoid receptor antagonist, has been shown to improve markers of renal and cardiovascular injury in patients with T2DM and CKD in both animal and phase 2 trials. In recent years, SGLT2i became the new standard therapy, the nonsteroidal corticosteroid receptor antagonist (MRA) finerenone has also been shown to bring definite cardiac and renal benefits to these patients. Based on new researches and evidences, the treatment recommendations in the 2022 KDIGO Guidelines (22) and the 2022 ADA Guidelines section”Chronic Kidney Disease and Risk Management” (9) have also been updated as follows: To delay renal disease progression and cardiovascular events, finerenone is recommended in people at high risk for renal disease progression and cardiovascular events or in patients who are unable to use SGLT2i and T2DM with CKD.

There are some cross and different pathophysiological pathways between finerenone and SGLT2i, and the combination of the two may achieve the effect superposition of cardiac and renal benefits through mechanism complementarity. The benefit of SGLT2i in delaying the progression of renal disease is independent of hypoglycemia. Although the relevant mechanism of action has not been fully elucidated, it is believed that SGLT2i may be related to the hemodynamic effect and the reduction of proteinuria. The mechanism of action of finerenone is mainly to reduce inflammation and fibrosis, reduce oxidative stress and improve endothelial function by inhibiting the excessive activation of salocorticoid receptor (MR), thus playing a role in reducing the progression of kidney disease. The different mechanisms of SGLT2i and finerenone complement each other, providing a theoretical basis for their combination. Finerenone provide an alternative treatment option for T2DM patients with CKD or CVD, with sustained cardiovascular benefits independent of or in combination with SGLT2i or glucagon-like peptide-1 receptor agonists (GLP-1RA). Head-to-head studies using finerenone and SGLT2i in patients with CKD or CVD are currently underway. The CONFIDENCE study may provide additional data on whether the combination of finerenone and SGLT2i produces stronger cardiovascular and renal protective effects compared to these drugs alone (23). Sotagliflozin has dual inhibitory effects on SGLT1 and SGLT2. The inhibition of SGLT1 can delay the absorption of glucose and galactose in digestive tract, while the inhibition of SGLT2 can reduce the reabsorption of glucose by renal tubules. In SOLOIST-WHF (20) and SCORED (19), sotagliflozin continued the previous improvement of SGLT2i in heart failure and prognosis, and significantly reduced the risk of MACE in DKD patients. In addition, in previous COVTs studies of SGLT2i (empagliflozin, canagliflozin, dapagliflozin, and ertugliflozin) showed no benefit for a single secondary endpoint (such as MI or NS), whereas in SCORED studies, sotagliflozin showed a significant reduction in the incidence of MI, NS by 32% and 34%. The effect of sotagliflozin on reducing MACE events was stronger than that of other SGLT2i, which may be related to the inhibitory effect of SGLT1 (24). The combination of finerenone and SGLT2i may be increased cardiovascular and renal survival benefits.

The definition of composite endpoints was slightly different in the trial. Some included studies were CVOTs (EMPA-REG, CANVAS, DECLARE-TIMI 58, VERTIS-CV), and renal outcomes were reported as secondary endpoints, which resulted in the inclusion of some patients with confirmed kidney disease or the exclusion of patients with severe kidney disease. In the studies conducted by SOLOIST-WHF, SCORED, FIGARO-DKD and FIDELIO-DKD, subjects were included in the eGFR of 25-90 mL/min/1.73m². CREDENCE included subjects eGFR 30-90 mL/min/1.73m². In addition, the subgroup analysis of the current FIDELIO-DKD study (by eGFR and UACR at baseline) reported cardiovascular outcomes including time to first HHF, the composite of time to CV death or first HHF (25), the data is not comprehensive. In the future, as more data are reported, the comparison of the protective effects of SGLT2i and finerenone on cardiovascular and renal outcomes of CKD stage III and above will continue to be concerned and further explored.

The limitation of this review is that although both SGLT2i and finerenone can reduce the occurrence of CV events, the external generalization ability of RCTs is limited due to certain statistical and subject enrollment limitations. We also note that these are large randomized controlled trials conducted in tightly controlled environments that do not necessarily reflect real-world conditions. The average follow-up time of the included studies was 2.9 years. The DECLARE-TIMI 58 follow-up time was longer than that of other studies, so there may have been unknown differences in cardiac and renal outcomes compared to other studies.

## Conclusion

In conclusion, this network meta-analysis demonstrated the effects of finerenone and SGLT2i (empagliflozin, canagliflozin, dapagliflozin, ertugliflozin, sotagliflozin) on cardiovascular and renal outcomes in patients with T2DM. Both SGLT2i and finerenone could reduce the risk of MACE, HHF, MI, CVD and RCO in varying degrees. Finerenone has no obvious advantage than SGLT2i on the effects of cardiovascular and renal protective. These findings will provide some evidence for the prevention and protection of T2DM patients with CKD or DKD.

## Data availability statement

The original contributions presented in the study are included in the article/**Supplementary Material**. Further inquiries can be directed to the corresponding author.

## Author contributions

XL and HP conceived and designed the study. HW and HP extracted information. XL and HW analyzed the data and wrote the manuscript. HP, and HJ reviewed the manuscript. All authors approved the final manuscript for submission.
