# Supplementary material for: Comparison the effects of finerenone and SGLT2i on cardiovascular and renal outcomes in patients with type 2 diabetes mellitus: A network meta-analysis
Source: Front Endocrinol (Lausanne). 2022 Dec 15;13:1078686. doi: 10.3389/fendo.2022.1078686 (PMC9797657; doi:10.3389/fendo.2022.1078686)
Supplement: Supplementary file 1 [file DataSheet_1.docx]

**Supplementary Table 1 Search strategies**

| Databases | Search strategies |
| --- | --- |
| PubMed | ("Finerenone"[Title/Abstract] OR "bay 94 8862"[Title/Abstract] OR "Empagliflozin"[Title/Abstract] OR "Canagliflozin"[Title/Abstract] OR "Dapagliflozin"[Title/Abstract] OR "Ertugliflozin"[Title/Abstract] OR "Sotagliflozin"[Title/Abstract] OR "SGLT-2i"[Title/Abstract] OR "sodium glucose cotransporter 2 inhibitors"[Title/Abstract]) AND ("randomized controlled study"[Title/Abstract] OR "RCT"[Title/Abstract]) AND ("diabetes mellitus, type 2"[MeSH Terms] OR "type 2 diabetes"[Title/Abstract] OR "diabetes type 2"[Title/Abstract] OR "diabetes mellitus type ii"[Title/Abstract] OR "noninsulin dependent diabetes mellitus"[Title/Abstract]) 45 |
| Embase | (Finerenone:ab,ti OR Empagliflozin:ab,ti OR Canagliflozin:ab,ti OR Dapagliflozin:ab,ti OR Ertugliflozin:ab,ti OR Sotagliflozin:ab,ti OR SGLT-2i:ab,ti OR Sodium-Glucose Cotransporter-2 Inhibitors:ab,ti) AND (diabetes:ab,ti OR 'diabetes mellitus':ab,ti OR 'type 2 diabetes mellitus':ab,ti OR dm:ab,ti OR t2dm:ab,ti) AND (Randomized controlled study:ab,ti OR RCT:ab,ti) AND（Cardiovascular Disease:ab,ti OR Diabetes kidney disease:ab,ti OR Renal outcome:ab,ti） |
| Cochrane Library | （Finerenone:ti,ab,kw OR Empagliflozin:ti,ab,kw OR Canagliflozin:ti,ab,kw OR Dapagliflozin:ti,ab,kw OR Ertugliflozin:ti,ab,kw OR Sotagliflozin:ti,ab,kw OR SGLT-2i:ti,ab,kw OR Sodium-Glucose Cotransporter-2 Inhibitors:ti,ab,kw）AND (Type 2 Diabetes:ti,ab,kw OR Diabetes, Type 2:ti,ab,kw OR Diabetes Mellitus, Type II:ti,ab,kw OR Noninsulin Dependent Diabetes Mellitus:ti,ab,kw) AND (Randomized controlled study:ti,ab,kw OR RCT:ti,ab,kw) AND（Cardiovascular Disease:ti,ab,kw OR Diabetes kidney disease:ti,ab,kw OR Renal outcome:ti,ab,kw） |

**Supplementary Table 2: An outline summary of the clinical trials**

| **Study** | **Study**  **Design** | **Inclusion criteria** | **Intervention** | **Primary Outcome** | **Secondary Outcome** |
| --- | --- | --- | --- | --- | --- |
| EMPA-REG | Multicentre randomized double-blind placebo-controlled trial | Insufficient glycemic control and high risk of CV events | Empagliflozin 10 mg, empagliflozin 25 mg or placebo | First occurrence of MACE (3-point) which included death from CV causes, non-fatal MI, or nonfatal stroke | Expanded occurrence of MACE to include unstable angina as well as HF exacerbation; renal events and transient ischemic attack |
| CANVAS | Randomized double-blind placebo-controlled | Male or female T2DM, ≥ 30 yrs with symptomatic CVD or 50 yrs or older with two or more RF for CVD | Canagliflozin 100 mg, canagliflozin 300 mg or placebo | Composite of death from CV causes;nonfatal MI  or nonfatal stroke; | Death from any cause  from CV cause;  progression of  albuminuria and  composite of death  from hospitalization for  HF; |
| CREDENCE | Randomized  double-blind  placebo-  controlled | T2DM with HbA1c ≥6.5 and ≤12% with eGFR ≥30 and ≤90, Pt. need to be on maximum tolerated dose of ACEi or ARB 4 weeks prior to randomization, UACR >300mg/g and <5000 mg/g | Canagliflozin 100mg or  Placebo | Composite of doubling of serum creatinine;ESRD and renal or CV death; | Composite Endpoint of  CV death and  Hospitalized HF; |
| DECLARE–TIMI 58 | Randomized  double-blind  placebo-  controlled | Male or female T2DM ≥ 40 yrs with T2DM and high risk for CV events | Dapagliflozin 10mg or  Placebo | Composite endpoint of  CV death;MI;Ischemic  stroke or ospitalization  due to HF; | Renal Composite  endpoint - ≥40%;  decrease in eGFR to  eGFR <60  ml/min/1.73m2 and/or  ESRD and/or Renal or  CV death; |
| VERTIS CV | Randomized  double-blind  placebo-  controlled | T2DM diagnosis, HbA1c 7-10.5%, on stable anti-hyperglycaemic  agents, BMI ≥18, hx of  atherosclerosis or  cerebro/peripheral vascular disease | Ertugliflozin 5mg, 15mg  or Placebo | Time to first MACE;  change from baseline in HbA1c% at week 18,  change from Haemoglobin baseline at week 18; | Time to occurrence of  CV death or HF  Hospitalization;  composite of renal  Death; renal  dialysis/transplant or  doubling serum  Creatinine; |
| SOLOIST-WHF | Randomized  double-blind  placebo-  controlled | 18-85yrs T2DM diagnosis;  symptoms of heart failure and received treatment  with intravenous diuretic therapy;eGFR＞30 mL/min/1.73m² | Sotagliflozin 200 mg，400 mg  Placebo | Composite endpoint of  CV death; | Hospitalized HF;  Death from any cause  from CV cause;  Deaths from any cause； |
| SCORED | Randomized  double-blind  placebo-  controlled | T2DM ≥ 18 yrs;  HbA1c 7-10.5%;  eGFR 25-90 ml mL/min/1.73m²;  High risk for CV Events; | Sotagliflozin 200 mg，400 mg  Placebo | Composite endpoint of  CV death; | Hospitalized HF;  Death from any cause  from CV cause;  Deaths from any cause；  Renal Composite  Endpoint:≥50% in the eGFR from baseline for ≥30 days, long-term dialysis, renal transplan  tation, or sustained eGFR of <15 ml/min/1.73 m² for ≥30 days |
| FIGARO-DKD | Randomized  double-blind  placebo-  controlled | T2DM ≥ 18 yrs ACR 30-300 and eGFR 25-90mL/min/1.73m²of body-surface area or UACR 300-5000 and eGFR＜60 mL/min/1.73m² | finerenone 10mg, 20 mg or placebo | Composite endpoint of  CV death; hospitalization  due to heart failure or  due to HF; | kidney failure;a sustained decrease from baseline of at least 40% in the eGFR or death from renal causes; |
| FIDELIO-DKD | Randomized  double-blind  placebo-  controlled | T2DM ≥ 18 yrs ACR 30-300 and eGFR 25-90 ml mL/min/1.73m² or UACR 300-5000 and eGFR＜60 mL/min/1.73m² | finerenone 10mg, 20 mg or placebo | kidney failure; a sustained decrease from baseline of at least 40% in the eGFR, or death from renal causes; | Composite endpoint of  CV death, ospitalization  due to heart failure or  due to HF; |

RCT=Randomized Controlled Trial, MACE=Major Adverse Cardiovascular Events, CVD=Cardiovascular Disease, RF=Risk Factors, MI= Myocardial Infarction, HF= Heart Failure, eGFR=Estimated Glomerular Filtration Rate, ESRD=End Stage Renal Disease, BMI=Body Mass Index, ACEi=Angiotensin Converting Enzyme Inhibitors, ARB=Angiotensin Receptor Blockers, T2DM=Type 2 Diabetes Mellitus, HbA1c=Glycosylated Hemoglobin Type A1C

**Supplementary Table 3: Pairwise comparison results from network meta-analysis on MACE**

| Empagliflozin | 0.94 (0.67, 1.28) | 1.08 (0.75, 1.55) | 1.13 (0.78, 1.62) | 0.84 (0.60, 1.17) | 1.01 (0.72, 1.38) | 1.16 (0.89, 1.52) |
| --- | --- | --- | --- | --- | --- | --- |
| 1.07 (0.78, 1.49) | Canagliflozin | 1.15 (0.85, 1.59) | 1.21 (0.88, 1.66) | 0.89 (0.68, 1.18) | 1.08 (0.83, 1.40) | **1.24 (1.04, 1.50)** |
| 0.92 (0.64, 1.33) | 0.87 (0.63, 1.18) | Dapagliflozin | 1.05 (0.73, 1.50) | 0.77 (0.56, 1.07) | 0.93 (0.68, 1.27) | 1.08 (0.84, 1.38) |
| 0.89 (0.62, 1.29) | 0.83 (0.60, 1.13) | 0.96 (0.67, 1.37) | Ertugliflozin | 0.74 (0.54, 1.03) | 0.89 (0.65, 1.23) | 1.03 (0.80, 1.34) |
| 1.19 (0.86, 1.67) | 1.12 (0.85, 1.46) | 1.29 (0.94, 1.78) | 1.35 (0.97, 1.86) | Sotagliflozin | 1.20 (0.91, 1.58) | **1.39 (1.13, 1.70)** |
| 0.99 (0.72, 1.38) | 0.93 (0.71, 1.20) | 1.07 (0.79, 1.47) | 1.12 (0.82, 1.54) | 0.83 (0.63, 1.09) | Finerenone | 1.16 (0.96, 1.39) |
| 0.86 (0.66, 1.13) | **0.80 (0.67, 0.97)** | 0.93 (0.73, 1.19) | 0.97 (0.75, 1.25) | **0.72 (0.59, 0.88)** | 0.87 (0.72, 1.04) | Placebo |

**Supplementary Table 4: Pairwise comparison results from network meta-analysis on MI**

| Empagliflozin | 0.98 (0.49, 1.97) | 1.02 (0.53, 2.01) | 1.19 (0.61, 2.33) | 0.78 (0.39, 1.58) | 1.03 (0.56, 1.89) | 1.15 (0.71, 1.88) |
| --- | --- | --- | --- | --- | --- | --- |
| 1.02 (0.51, 2.04) | Canagliflozin | 1.05 (0.53, 2.05) | 1.22 (0.62, 2.42) | 0.80 (0.40, 1.62) | 1.06 (0.57, 1.94) | 1.18 (0.72, 1.92) |
| 0.98 (0.50, 1.90) | 0.96 (0.49, 1.88) | Dapagliflozin | 1.17 (0.60, 2.27) | 0.76 (0.39, 1.53) | 1.01 (0.56, 1.81) | 1.12 (0.71, 1.79) |
| 0.84 (0.43, 1.65) | 0.82 (0.41, 1.62) | 0.86 (0.44, 1.66) | Ertugliflozin | 0.66 (0.33, 1.32) | 0.86 (0.47, 1.59) | 0.96 (0.60, 1.54) |
| 1.28 (0.63, 2.57) | 1.25 (0.62, 2.52) | 1.31 (0.65, 2.58) | 1.52 (0.76, 3.04) | Sotagliflozin | 1.32 (0.70, 2.46) | 1.47 (0.89, 2.44) |
| 0.97 (0.53, 1.79) | 0.95 (0.52, 1.76) | 0.99 (0.55, 1.80) | 1.16 (0.63, 2.11) | 0.76 (0.41, 1.43) | Finerenone | 1.11 (0.77, 1.62) |
| 0.87 (0.53, 1.42) | 0.85 (0.52, 1.39) | 0.89 (0.56, 1.41) | 1.04 (0.65, 1.66) | 0.68 (0.41, 1.13) | 0.90 (0.62, 1.29) | Placebo |

**Supplementary Table 5: Pairwise comparison results from network meta-analysis on** **HHF**

| Empagliflozin | 0.98 (0.60, 1.63) | 1.12 (0.64, 1.97) | 1.08 (0.6, 1.93) | 1.01 (0.61, 1.67) | 1.20 (0.73, 1.97) | **1.54 (1.02, 2.32)** |
| --- | --- | --- | --- | --- | --- | --- |
| 1.02 (0.61, 1.68) | Canagliflozin | 1.14 (0.71, 1.84) | 1.09 (0.66, 1.80) | 1.03 (0.68, 1.53) | 1.22 (0.81, 1.83) | **1.56 (1.17, 2.09)** |
| 0.89 (0.51, 1.56) | 0.88 (0.54, 1.41) | Dapagliflozin | 0.96 (0.55, 1.67) | 0.90 (0.57, 1.45) | 1.07 (0.67, 1.71) | 1.37 (0.94, 2.00) |
| 0.93 (0.52, 1.66) | 0.92 (0.56, 1.51) | 1.04 (0.60, 1.82) | Ertugliflozin | 0.94 (0.57, 1.55) | 1.12 (0.68, 1.84) | 1.43 (0.95, 2.16) |
| 0.99 (0.60, 1.64) | 0.97 (0.65, 1.46) | 1.11 (0.69, 1.77) | 1.06 (0.64, 1.76) | Sotagliflozin | 1.19 (0.80, 1.77) | **1.52 (1.15, 2.02)** |
| 0.83 (0.51, 1.37) | 0.82 (0.55, 1.24) | 0.93 (0.58, 1.50) | 0.89 (0.54, 1.47) | 0.84 (0.56, 1.24) | Finerenone | 1.28 (0.97, 1.70) |
| **0.65 (0.43, 0.98)** | **0.64 (0.48, 0.86)** | 0.73 (0.50, 1.06) | 0.70 (0.46, 1.05) | **0.66 (0.50, 0.87)** | 0.78 (0.59, 1.03) | Placebo |

**Supplementary Table 6: Pairwise comparison results from network meta-analysis on CVD**

| Empagliflozin | 1.34 (0.86, 2.09) | 1.58 (0.96, 2.58) | 1.48 (0.90, 2.43) | 1.42 (0.89, 2.25) | 1.42 (0.91, 2.21) | **1.61 (1.12, 2.32)** |
| --- | --- | --- | --- | --- | --- | --- |
| 0.75 (0.48, 1.16) | Canagliflozin | 1.18 (0.77, 1.80) | 1.11 (0.73, 1.71) | 1.06 (0.72, 1.54) | 1.06 (0.74, 1.52) | 1.20 (0.93, 1.56) |
| 0.63 (0.39, 1.04) | 0.85 (0.56, 1.30) | Dapagliflozin | 0.94 (0.58, 1.52) | 0.90 (0.58, 1.39) | 0.90 (0.59, 1.37) | 1.02 (0.73, 1.43) |
| 0.68 (0.41, 1.11) | 0.90 (0.59, 1.38) | 1.06 (0.66, 1.73) | Ertugliflozin | 0.96 (0.61, 1.48) | 0.96 (0.62, 1.45) | 1.09 (0.77, 1.53) |
| 0.70 (0.44, 1.12) | 0.94 (0.65, 1.39) | 1.11 (0.72, 1.74) | 1.04 (0.67, 1.63) | Sotagliflozin | 1.00 (0.69, 1.46) | 1.14 (0.86, 1.51) |
| 0.70 (0.45, 1.10) | 0.94 (0.66, 1.34) | 1.11 (0.73, 1.70) | 1.04 (0.69, 1.61) | 1.00 (0.68, 1.45) | Finerenone | 1.13 (0.89, 1.46) |
| **0.62 (0.43, 0.89)** | 0.83 (0.64, 1.07) | 0.98 (0.70, 1.37) | 0.92 (0.65, 1.29) | 0.88 (0.66, 1.16) | 0.88 (0.69, 1.13) | Placebo |

**Supplementary Table 7: Pairwise comparison results from network meta-analysis on NS**

| Empagliflozin | 0.73 (0.35, 1.49) | 0.81 (0.40, 1.66) | 0.81 (0.39, 1.68) | 0.53 (0.25, 1.12) | 0.81 (0.43, 1.53) | 0.81 (0.48, 1.37) |
| --- | --- | --- | --- | --- | --- | --- |
| 1.38 (0.67, 2.84) | Canagliflozin | 1.12 (0.57, 2.26) | 1.11 (0.54, 2.27) | 0.73 (0.36, 1.52) | 1.11 (0.61, 2.07) | 1.11 (0.68, 1.83) |
| 1.23 (0.60, 2.51) | 0.89 (0.44, 1.76) | Dapagliflozin | 0.99 (0.49, 2.00) | 0.65 (0.32, 1.33) | 0.99 (0.54, 1.82) | 0.99 (0.61, 1.59) |
| 1.24 (0.60, 2.58) | 0.90 (0.44, 1.84) | 1.01 (0.50, 2.05) | Ertugliflozin | 0.66 (0.31, 1.38) | 1.00 (0.54, 1.89) | 1.00 (0.60, 1.69) |
| 1.88 (0.89, 3.99) | 1.36 (0.66, 2.81) | 1.53 (0.75, 3.13) | 1.52 (0.72, 3.2) | Sotagliflozin | 1.51 (0.80, 2.89) | 1.52 (0.89, 2.60) |
| 1.24 (0.65, 2.34) | 0.90 (0.48, 1.65) | 1.01 (0.55, 1.84) | 1.00 (0.53, 1.86) | 0.66 (0.35, 1.24) | Finerenone | 1.00 (0.69, 1.43) |
| 1.24 (0.73, 2.09) | 0.90 (0.55, 1.47) | 1.01 (0.63, 1.64) | 1.00 (0.59, 1.67) | 0.66 (0.38, 1.12) | 1.00 (0.70, 1.44) | Placebo |

**Supplementary Table 8: Pairwise comparison results from network meta-analysis on RCO**

| Empagliflozin | 1.08 (0.60, 1.91) | 0.87 (0.45, 1.68) | 1.33 (0.69, 2.63) | 1.16 (0.56, 2.43) | 1.38 (0.81, 2.42) | **1.64 (1.04, 2.57)** |
| --- | --- | --- | --- | --- | --- | --- |
| 0.93 (0.52, 1.66) | Canagliflozin | 0.80 (0.45, 1.48) | 1.23 (0.69, 2.27) | 1.08 (0.55, 2.14) | 1.28 (0.82, 2.09) | **1.51 (1.09, 2.17)** |
| 1.15 (0.59, 2.24) | 1.25 (0.68, 2.24) | Dapagliflozin | 1.53 (0.78, 3.06) | 1.34 (0.63, 2.85) | 1.59 (0.91, 2.86) | **1.89 (1.17, 3.08)** |
| 0.75 (0.38, 1.45) | 0.81 (0.44, 1.45) | 0.65 (0.33, 1.28) | Ertugliflozin | 0.87 (0.40, 1.88) | 1.04 (0.57, 1.86) | 1.23 (0.75, 1.99) |
| 0.86 (0.41, 1.80) | 0.93 (0.47, 1.81) | 0.75 (0.35, 1.60) | 1.14 (0.53, 2.51) | Sotagliflozin | 1.19 (0.61, 2.34) | 1.41 (0.77, 2.56) |
| 0.72 (0.41, 1.24) | 0.78 (0.48, 1.22) | 0.63 (0.35, 1.10) | 0.96 (0.54, 1.74) | 0.84 (0.43, 1.63) | Finerenone | 1.19 (0.85, 1.62) |
| **0.61 (0.39, 0.96)** | **0.66 (0.46, 0.92)** | **0.53 (0.32, 0.85)** | 0.81 (0.50, 1.34) | 0.71 (0.39, 1.29) | 0.84 (0.62, 1.17) | Placebo |

**Supplementary Table 9:** **SUCRA ranking table for cardiovascular and renal outcomes**

|  | **MACE** | **MI** | **HHF** | **CVD** | **NS** | **RCO** |
| --- | --- | --- | --- | --- | --- | --- |
| **Canagliflozin** | 0.7349375 | 0.5852792 | 0.7284375 | 0.6453250 | 0.6187208 | 0.6344292 |
| **Dapagliflozin** | 0.3581667 | 0.5155542 | 0.4739875 | 0.2789417 | 0.4362208 | 0.8841000 |
| **Empagliflozin** | 0.5571708 | 0.5446792 | 0.6849000 | 0.9563875 | 0.1742417 | 0.7350875 |
| **Ertugliflozin** | 0.2469833 | 0.2458250 | 0.5586708 | 0.4162167 | 0.4552417 | 0.3556208 |
| **Sotagliflozin** | 0.9297417 | 0.8524792 | 0.6871250 | 0.5128458 | 0.9112750 | 0.5291500 |
| **Finerenone** | 0.5489708 | 0.4976333 | 0.3435542 | 0.5169583 | 0.4550542 | 0.2995625 |
| **Placebo** | 0.1240292 | 0.2585500 | 0.0233250 | 0.1733250 | 0.4492458 | 0.0620500 |
